# Ascorbic acid has superior antiviral and antiproliferative effects over IFN-alpha in HAM/TSP PBMC ex vivo

**DOI:** 10.1186/1742-4690-8-S1-A61

**Published:** 2011-06-06

**Authors:** Britta Moens, Daniele Decanine, Ricardo Khouri, Giovanni Lopez, Michael Talledo, Eduardo Gotuzzo, Françoise Bex, Bernardo Galvão Castro, Anne-Mieke Vandamme, Johan Van Weyenbergh

**Affiliations:** 1 Laboratory of Clinical and Epidemiological Virology, Rega Institute for Medical Research K.U. Leuven, Leuven, Belgium; 2 Gonçalo Moniz Research Center, Oswaldo Cruz Foundation (FIOCRUZ), Salvador-Bahia, Brazil; 3 Instituto de Medicina Tropical Alexander von Humboldt, Universidad Peruana Cayetano Heredia, Lima, Peru; 4 Departamento de Medicina, Facultad de Medicina, Universidad Peruana Cayetano Heredia, Lima, Peru; 5 Institute for Microbiological Research J-M Wiame and Laboratory of Microbiology, Université Libre de Bruxelles, Bruxelles, Belgium

## 

IFN-alpha and high dose ascorbic acid (AA) have a modest clinical benefit in HAM/TSP (Nakagawa, 1996). We investigated the effect of ex vivo and in vitro AA and IFN-alpha treatment on HAM/TSP PBMC and HTLV-1-infected cell lines, respectively.

We treated cells for 48-72h ex vivo and in vitro with low-high (10-100µg/ml) dose of AA and 1000U/ml of IFN-alpha and quantified lymphoproliferation by [^3^H]thymidine incorporation, tetraploid DNA content and PCNA expression (flow cytometry). Viral expression was measured at the RNA (tax, LTR) and protein (Tax, p19) level by RT-PCR, Western blot and ELISA, respectively. Apoptosis was quantified by subdiploid DNA content (flow cytometry). Th1/Th2/Th17 cytokines were quantified by cytometric bead array. AA induced a dramatic 95% decrease (control 3689±755 cpm vs. AA 121±52 cpm, p=0.001) in spontaneous, virus-driven lymphoproliferation and a decrease in tax and LTR transcription in HAM/TSP PBMC. In addition, AA decreased the exacerbated ex vivo IFN-gamma production in HAM/TSP PBMC. In HTLV-1 infected cell lines (MT-2 and MT-4), AA induced a dose-dependent increase in DNA fragmentation (p=0.02, p=0.005), paralleled by a decrease in PCNA (p=0.003), as well as p19 (p<0.001) and Tax levels. These effects appear virus-specific, since high-dose (100µg/ml) AA did not exert a significant antiproliferative or pro-apoptotic effect on PBMC of normal donors. On top, AA displayed a superior antiproliferative, antiviral and immunomodulatory effect over IFN-alpha in both cell lines and HAM/TSP PBMC. Considering the selective antiproliferative and antiviral effects of AA ex vivo and in vitro, the therapeutic potential of combination therapy with high dose AA in HAM/TSP should be further explored.
